# Can depression be a menopause-associated risk?

**DOI:** 10.1186/1741-7015-8-79

**Published:** 2010-12-01

**Authors:** Claudio N Soares

**Affiliations:** 1 Department of Psychiatry and Behavioural Neurosciences, McMaster University, Hamilton, Ontario, Canada; 2 Department of Obstetrics and Gynecology, McMaster University, Hamilton, Ontario, Canada; 3 Mood Disorders Division, McMaster University, Hamilton, Ontario, Canada

## Abstract

There is little doubt that women experience a heightened psychiatric morbidity compared to men. A growing body of evidence suggests that, for some women, the menopausal transition and early postmenopausal years may represent a period of vulnerability associated with an increased risk of experiencing symptoms of depression, or for the development of an episode of major depressive disorder. Recent research has begun to shed some light on potential mechanisms that influence this vulnerability. At the same time, a number of studies and clinical trials conducted over the past decade have provided important data regarding efficacy and safety of preventative measures and treatment strategies for midlife women; some of these studies have caused a shift in the current thinking of how menopausal symptoms should be appropriately managed.

Essentially, most women will progress from premenopausal into postmenopausal years without developing significant depressive symptoms. However, those with prior history of depression may face a re-emergence of depression during this transition while others may experience a first episode of depression in their lives. Here I provide an overview of what is known about risk factors for depression and the risk posed by the menopausal transition, its associated symptoms, and the underlying changes in the reproductive hormonal milieu, discussing the evidence for the occurrence of mood symptoms in midlife women and the challenges that face clinicians and health professionals who care for this population.

## Introduction

Over the past few decades, growing epidemiological and clinical data support the notion that some, but not all, women may be at a heightened risk for psychiatric morbidity (for example, mood and anxiety symptoms, cognitive complaints) during periods in life that are associated with reproductive cycle events such as the postpartum period and the menopausal transition. These periods are not only marked by extreme hormone variations but may also be accompanied by the occurrence of significant life stressors and changes in personal, family, and professional responsibilities [1]. The complexity of the so-called female-specific 'windows of vulnerability' certainly poses a particular challenge to physicians and other professionals dedicated to women's health issues across the life span.

**Figure 1:**
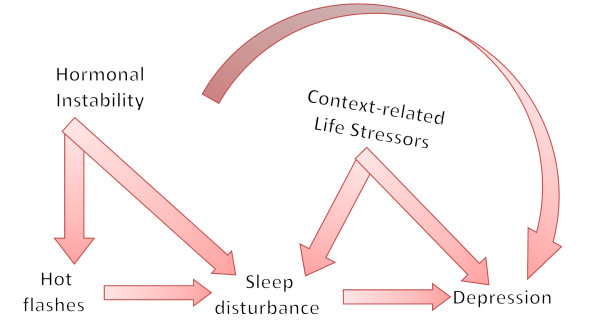
**Exploring pathways for menopause-associated depression **.

Among these female-specific 'windows of vulnerability', the menopausal transition constitutes a complex example: this transition is marked by progressive, dynamic changes in sex hormones and reproductive function. At the same time, these changes overlap with the aging process *per se*, and with modifications in metabolism, sexuality, lifestyle behaviors and overall health [2]. During this period in life, some individuals may seek medical treatment for a constellation of symptoms including vasomotor complaints (VMS; that is, hot flashes, night sweats), aches and stiff joints, trouble sleeping, and lack of energy. Others, on a more preventative approach, will inquire about different strategies (for example, hormonal, non-hormonal, 'natural' remedies) to promote a 'healthier' transition into postmenopausal years. Overall, diagnostic and therapeutic approaches to symptomatic women during the menopausal transition and postmenopausal years are, or should be, multifaceted and multidisciplinary in nature (Figure 1).

## Discussion

For decades, the existence of a more causal, direct association between the menopausal transition and the emergence of depressive symptoms has been the subject of intense controversy; the question quite often posed by researchers and clinicians was whether the occurrence of menopause-associated depression was caused primarily by stressful life events, psychological factors or by the impact of ovarian hormonal changes. This controversy is illustrated by the array of different theories and methodological approaches that has guided most of the research in this field. Some cross-sectional studies examining the relationship between menopause and depressive symptoms revealed no association [3-6], while others showed an increase in depression among women in the menopausal transition [7,8]. Among various theories, some have postulated that the occurrence of depression during this time in life could be in part modulated by the presence of sleep disruption and/or the occurrence of VMS. However, a recent study investigated this hypothesis by exploring the interactions between sleep, vasomotor symptoms and depression using objective and subjective sleep parameters in midlife women with and without depression [9]. Although depressed women reported poorer sleep quality and efficiency than non-depressed women, the two groups did not differ with respect to measurements of sleep interruption. Further, there was, no increased frequency of nocturnal vasomotor symptoms or awakenings observed among depressed women, refuting a direct link hypothesis.

Unlike the cross-sectional studies, most prospective studies [10-14] have systematically confirmed the menopausal transition as a period of heightened risk for development of depressive symptoms and/or depression. The Penn Ovarian Aging Study [12] showed an increased risk for depressive symptoms during the transition to menopause followed by a decrease in this risk in subsequent years (that is, in the postmenopausal period); the authors also suggested that depression and hormone-related symptoms could share some underlying mechanisms since history of severe PMS (premenstrual syndrome), emergence of hot flashes and sleep problems were independent predictors of depression in this population. Two long-term prospective studies followed women with no history of depression across the menopause transition to examine the risk for new onset of depression [15,16]. In both studies, a 6-8-year follow-up of premenopausal women (n = 460 and n = 231, respectively) revealed a significant increased risk (twofold to fourfold) for developing depression in women as they entered perimenopause compared to those who remained premenopausal. Moreover, greater variation of estradiol and follicle-stimulating hormone (FSH) levels over the follow-up period appear to be associated with higher depressive scores and diagnosis of MDD; an indicator that wide fluctuation in hormonal levels, rather than their absolute levels, may be a contributing factor to the emergence of depression in biologically vulnerable women. Other mediators of the risk for depression in this particular population include ethnicity (higher risk in African American, lower risk in Asian population), lower education, past history of postpartum blues or postpartum depression, cigarette smoking, stressful life events [10-16].

As highlighted above, epidemiologic evidence supports the 'critical timing' hypothesis for the occurrence of depression, most likely linked to periods of hormone instability rather then hormone deficiency. Interestingly, different areas of medicine exploring health-related outcomes in midlife and aging women seem to agree on the same notion of a 'critical timing window' being associated with the menopausal transition and the importance of proper timing for preventive or therapeutic strategies. For example, recent evidence from the Study of Women's Health Across the Nation (SWAN) showed changes in processing speed and verbal episodic memory in women during the menopausal transition, with memory performance returning to premenopausal levels in the postmenopausal years [17]. Changes in estrogen levels during the menopausal transition have also been associated with heightened risk for adverse cardiovascular events, including larger adventitial carotid artery diameters, changes in lipids, and increased blood pressure [18,19].

Evidence from clinical trials supports the concept of a window of opportunity for hormonal treatment strategies in midlife women, not only for cardiovascular diseases but also, as further discussed, for menopause-associated depression. An age-stratified analysis of the Women's Health Initiative (WHI) data revealed that several of the adverse events initially associated with menopausal hormone therapies (MHTs) in the entire study population were not evident in the subgroup of younger, postmenopausal women (between 50 and 59 years of age) or among those women who had recently transitioned into menopause. In fact, there seemed to be a cardioprotective effect of MHT (particularly estrogen based) when hormone therapies were administered to women within 10 years of the final menstrual period, possibly because of the fewer preexisting coronary conditions in this subgroup [20]. Further studies are needed to explore the risks and benefits of hormone therapies in younger, symptomatic women. Large-scale clinical studies such as Early versus Late Intervention Trial with Estrogen (ELITE; NCT00114517) and Kronos Early Estrogen Prevention Study (KEEPS; NCT00154180) [21] should be instrumental for the evaluation of 'critical timing' for the use of hormone strategies from a cardiovascular and a cognitive perspective.

Antidepressant agents and psychotherapeutic treatments are widely accepted as first-line treatments for depression across the life span. Indeed, several trials have documented the efficacy of psychotropic medications for the alleviation of mood and other menopause-related symptoms in perimenopausal and postmenopausal women [22-26]. Despite that, preclinical and clinical evidence supports a possible window of opportunity for hormonal treatments to manage depressive episodes associated with the menopausal transition; after all, estrogen can, in many ways, modulate systems that are critical for mood and behavior regulation, particularly through pathways involving monoaminergic neurotransmission (serotonin (5-HT), norepinephrine (NE)) [27].

Estradiol (E2) administration decreases the activity of monoamine oxidases (MAO-A and MAO-B), which are enzymes involved in 5-HT degradation; E2 administration also increases both isoforms of tryptophan hydroxylase (TPH), the rate-limiting enzyme of serotonin synthesis, resulting in an overall increase in 5-HT synthesis and availability. Furthermore, by downregulating 5HT_1a _autoreceptors and upregulating 5HT_2a _receptors, E2 increases the amount of serotonin found in the synapse and increases the amount available for postsynaptic transmission. Similarly, estrogens increase NE availability by decreasing expression of MAOs and increasing the activity of tyrosine hydroxylase, the rate-limiting enzyme in the synthetic pathway of catecholamine [28].

Estrogen-based therapies have shown superior antidepressant effects compared to placebo when administered to perimenopausal women [29,30]. The antidepressant effects of estrogen were observed even in the absence of concomitant vasomotor symptoms [30]. Older, postmenopausal women, however, showed little or no response to estrogen therapy for the alleviation of depressive symptoms [31]. Taken together, these observations suggest that estrogen's antidepressant effect may have a 'critical window' or optimal timing, possibly during the MT and early postmenopausal years; moreover, the potential benefits of E2 therapy for the improvement of mood symptoms may occur independent from changes/improvement of vasomotor symptoms.

As symptomatic midlife women may endorse a wide variety of physical and emotional complaints, physicians and other health professionals have the challenging task to disentangle the biological and psychosocial aspects that could be primarily attributed to (a) the aging process *per se*; (b) the menopausal transition; (c) the emergence of new onset/recurrent depression.

## Conclusions

Robust epidemiologic evidence supports the existence of a 'window of vulnerability' for the occurrence of depression (new onset or recurrent) during the menopausal transition and early postmenopausal years. Similar to that observed in other periods in life, antidepressants and psychotherapies continue to be the treatments of choice for depression occurring in midlife women. Nonetheless, accumulated preclinical and clinical data support the benefits of estrogen-based therapies to improve mood and other menopause-related symptoms during this critical window. As such, estrogen should be considered part of the treatment armamentarium for depression, along with other well established benefits (for example, for vasomotor, sexual and other menopause-related complaints) [32]. Lastly, the use of specific screening and diagnostic tools for depression in women [33] should help physicians and health professionals to improve early detection and clinical management. Ultimately, treatment strategies should be tailored and incorporate all the resources available to reduce the significant burden and functional impairment associated with depression in women undergoing menopause.

## Competing interests

CNS has received research support from Allergen National Centre of Excellence, the Canadian Institutes of Health Research, the NARSAD Brain and Behavior Research Foundation, Eli Lilly, Pfizer, AstraZeneca, and Hamilton Community Foundation. He has received honoraria as a consultant and/or speaker for Wyeth, Pfizer, AstraZeneca, Eli Lilly, Lundbeck, and Bayer Schering Pharmaceuticals.

## Pre-publication history

The pre-publication history for this paper can be accessed here:

http://www.biomedcentral.com/1741-7015/8/79/prepub
